# Recommendations for the quantitative basic evaluation of mind-body medicine courses for university students – results of a consensus-based, systematic decision-making process by experts

**DOI:** 10.1186/s12909-024-06387-x

**Published:** 2024-11-27

**Authors:** Daniela Adam, Peter Vogelsänger, Benno Brinkhaus, Barbara Stöckigt

**Affiliations:** 1 Institute of Social Medicine, Epidemiology and Health Economics, Charité Universitätsmedizin Berlin, Corporate Member of Freie Universität Berlin, Humboldt-Universität Zu Berlin, and Berlin Institute of Health, Berlin, Germany; 2 Institute for General Medicine, University Hospital, Otto-Von-Guericke-Universität Magdeburg, Magdeburg, Germany

**Keywords:** Mind–body medicine, Medical education, University students, Quantitative evaluation, Learning objectives, Iterative process, Decision-making process

## Abstract

**Background:**

There is an increasing range of mind–body medicine (MBM) courses in Germany to support university students in dealing with stress. The evaluation of these varies and often only has a small number of participants due to the limited group size of the courses. The aim of this project was the development of a quantitative basic evaluation that can be used across all sites that conduct MBM courses.

**Methods:**

In a consensus-based and systematic decision-making process, the learning objectives and various questionnaires for the evaluation of MBM courses were discussed and evaluated by experts according to defined criteria. The process was iterative, in which the reflection and definition of the learning objectives and the questionnaires were conditional and adapted if necessary. The recommendations for the basic evaluation of students’ MBM courses were developed by consensus among the experts.

**Results:**

For the experts, the most important learning objectives of the MBM courses were stress reduction and self-experience with the exercises. A total of 21 questionnaires were evaluated from nine topics: mindfulness, empathy, self-reflection, self-efficacy, resilience, stress, sense of coherence, quality of life, and well-being. Finally, eight questionnaires were recommended by the expert group for use in a basic evaluation: stress (PSS-10), quality of life (WHOQOL-Bref), mindfulness (MAAS), self-efficacy (GSE), self-compassion (SCS), empathy (SPF), self-reflection (GRAS) and sense of coherence (SOC-13). Further questionnaires were recommended as "optional". An additional qualitative evaluation is recommended for a broader and deeper understanding of the quantitative results.

**Conclusions:**

The proposed basic evaluation is the central result of the iterative consensus-based decision-making process, which reflects the learning objectives of the underlying MBM courses. We hope that the basic evaluation will be integrated into other MBM courses so that results of various courses can be pooled and compared across sites in the future. This could increase the informative value of the evaluations. Furthermore, researchers could consider the use of the basic evaluation in clinically controlled trials on MBM.

**Trial registration:**

The project was not registered in a clinical trial registry because no results from health care interventions on human participants have been analyzed or reported.

## Background

In Germany, an increase in stress-related diseases can be observed, particularly among medical students [1]. The high demands that each new generation of medical students face during their university careers is a considerable strain to their mental and physical health [2–4]. This has serious consequences for those who are affected and can impact their later professional careers. Stressed or even depressed doctors are less empathetic and lack concentration. They are six times more likely to make treatment errors than non-depressed colleagues [5]. As a result, some faculties teach their students how to deal better with stress through mind–body medicine (MBM) methods before they start their careers and to consolidate their self-care. MBM is part of Complementary and Integrative Medicine and focuses on the interaction between brain, mind, body and behavior and its effects on health [6]. This has led to an increasing number of MBM courses being offered to students at German universities and colleges [3, 7–9]. These are usually optional or elective courses. Their learning objectives, content and methods may vary from faculty to faculty. Gebauer et al. mentioned in this context that there is room for improvement in the critical evaluation of these courses and cooperation between universities [10].

Some of the MBM courses are scientifically evaluated to measure possible effects and to continuingly develop them. A wide range of questionnaires and data collection tools is available to assess various constructs, such as self-efficacy, mindfulness, stress, and resilience. The group size in the courses is often small for various reasons, such as maintaining a protected and confidential atmosphere, enabling group dynamics, and limited resources. Therefore, quantitative evaluation results are often of limited significance if they have not been gathered continuingly over several years. The use of additional qualitative evaluation methods often allows a broader and deeper understanding of the quantitative results [3].

The problem of the limited significance of quantitative data led to the aim of developing a quantitative basic evaluation, i.e., to create a set of questionnaires that can be used at various sites offering MBM courses for students to prospectively enable the pooling of results and thus increase the informative value of the evaluations.

Furthermore, there are clear indications that too many questionnaires have a negative impact on the quality of completion [11, 12]. Therefore, an additional objective was to take a critical look at the evaluation instruments used to date and, if necessary, improve and streamline them.

## Methods

Various questionnaires for the evaluation of MBM courses were gathered and discussed by experts in a consensus-based and systematic decision-making process. The panel of experts comprised four members, one medical scientist and three physicians, including specialists in psychosomatic medicine and psychotherapy, internal medicine and an expert in epidemiological and qualitative research. All the experts have many years of expertise in MBM as well as at least five years of teaching experience in respective courses. The process was iterative, in which the reflection and definition of the learning objectives and the questionnaires for the course evaluation were conditional and adapted if necessary. The members of the expert panel are the authors of this article. All the experts worked voluntarily in this project. No ethics approval or written informed consent was required as our institutional review board, the “Ethikkommission der Medizinischen Fakultät Charité – Universitätsmedizin Berlin”, does not advise any research which does not include human beings or human material, nor personal data.

No formal consensus development method [13] (e.g. the Delphi method, the nominal group technique, or a consensus development conference) was used to address the research questions. This has various reasons. First, the consensus-based, iterative process bypassed some of the main limitations of formal consensus development methods [13–15]. The consensus-based and systematic decision-making process within the relatively small expert panel enabled lively discussion between the experts, direct responses have been obtained and no voting process was needed. The research paradigm of this work could be considered to be a constructivist analysis. Constructivists suppose that knowledge exists within the self and is constructed by individuals as they interact with themselves and others [16]. Furthermore, the process was realized with little financial and timely resources. Nonetheless, our applied method, the iterative, consensus-based and systematic decision-making process, has limitations as well, which will be addressed carefully in the discussion section.

In the run-up to the project, the expert panel met five times between February and October 2021 to compare the courses, their exercises and evaluation methods as well as their structural embedding in the medical faculties of the university hospitals Berlin and Magdeburg to clarify differences and similarities. The meetings lasted approximately 1 to 1.5 h each, and minutes were taken.

### Iterative process

Due to the iterative process, methods and results are interdependent. First, the learning objectives of the MBM courses were updated and summarized. This included objectives that had already been evaluated as well as new objectives. Second, the questionnaires used to date were categorized by topic, according to the course objectives. The search for possible additional questionnaires that had not yet been used was carried out unsystematically via the internet and was based on the experts' prior knowledge. Systematic analysis criteria were developed and applied to evaluate the questionnaires: advantages and disadvantages in terms of costs and user fees, number of items, comprehensibility of the items and the questionnaire as a whole, wording of the questions or items (e.g., positively or negatively worded), frequency of use (number of PubMed hits in February 2024), and availability in different languages. Additionally, the experts could make further comments on the questionnaires. All questionnaires were assessed in a structured manner by all experts between November 2021 and August 2022. In total, the panel met seven times during this period for approximately 1.5 h each time. Between the meetings, individual members of the panel took on defined tasks, which were then discussed and finally agreed upon at the following meetings, e.g., topic-related internet research and individual examination of certain questionnaires.

The questionnaires were selected by consensus after intensive discussion, consideration and weighting. A standardized assessment, for example, by distributing points, was not carried out. As a result, the questionnaires were rated as "recommended", "optional" or "rather not recommended".

The interim results and underlying concepts of the topics were continuingly reflected, discussed and defined in the group. The results, i.e., the compiled basic evaluation (all recommended questionnaires), were tested for applicability by all experts.

## Results

### Objectives of the MBM courses

As part of the iterative discussion in the expert panel, a process of reflection on the already established course objectives and their evaluation was initiated. The central questions were: What are the learning objectives of the courses? Why do we consider the use of certain questionnaires to be essential for the evaluation of these objectives or not?

The learning objectives of the MBM courses were then updated respectively: stress reduction, practical self-experience with MBM exercises, a mindful and self-caring attitude, self-reflection, strengthening of communication skills, supporting student health, and learning methods for stress reduction to apply them together with patients in the later professional practice.

### Selection and assessment of the questionnaires

Table 1 summarizes the results of the evaluation process of various questionnaires.

**Table 1:** Presentation of the instruments for quantitative evaluation of the MBM courses

Topic / Questionnaire	Content, Number of items	Advantages	Disadvantages	Recommendation
**Mindfulness**				
Freiburg Mindfulness Inventory (FMI) [17, 18]	Mindfulness, 14	• Positive wording of items • PubMed hits: 35 • Available in at least 8 languages	• Unclear separation of self-love and mindfulness	Rather not recommended
Mindful Attention and Awareness Scale (MAAS) [19]	Dispositional mindfulness, 15	• Applies to everyday life situations • Only aligned to mindfulness • PubMed hits: 222 • Available in at least 22 languages	• Negative wording of items	Recommended
Self Compassion Scale—Shortform (SCS) [20]	Self-love, Compassion and mindfulness, 12	• Positive and negative wording of items • PubMed hits: 166 • Available in at least 22 languages		Recommended
**Empathy**				
Interpersonal Reactivity Index (IRI) [21]	Multi-dimensional assessment of empathy, 28	• PubMed hits: 326 • Available in at least 5 languages	• Lengthy	Rather not recommended
Saarbrucken personality questionnaire (SPF) [22]	Empathy, short German version of the IRI, 16	• Simple scoring • Good parameters: reliability, validity, item selectivity	• No identical English version available but based on IRI • PubMed hits: 1	Recommended
Brief IRI (B-IRI) [23]	Empathy, 16		• No validated German version available • No further information on language availability • PubMed hits: 1	Rather not recommended
Jefferson Scale of Physician Empathy, Student Version (JSE) [24]	Empathy of medical doctors, 20	• PubMed hits: 77 • Available in at least 59 languages / dialects	• Restricted to empathy of medical doctors • Tendency to be rigid	Optional
**Self-reflection**				
Groningen Reflection Ability Scale (GRAS) [25]	Self-reflection, Empathy, 23	• Good differentiability from other topics / questionnaires • Available in at least 5 languages	• Strong mental focus • Lengthy • PubMed hits: 10	Recommended
**Self-efficacy**				
General Self-Efficacy Scale (GSE) [26]	Self-efficacy, 10	• PubMed hits: 214 • Available in at least 33 languages	• Predominantly problem-orientated (negative) wording of items	Recommended
General Self-Efficacy Scale (GSE 6) [27]	Self-efficacy, validated short-form of the GSE, 6	• Available in at least 2 languages	• Paid questionnaire • PubMed hits: 5	Rather not recommended
Short Scale for Measuring General Self-efficacy Beliefs (ASKU) [28]	Self-efficacy, 3	• Available in at least 2 languages	• Recently published (2021) • PubMed hits: 8	Optional
**Resilience**				
Resilience Scale (RS-13) [29]	Mental resistance, Self-awareness, Self-love,13		• Only German version available • Very similar to self-efficacy • PubMed hits: 11	Rather not recommended
Connor Davidson Resilience Scale-10 (CD-RISC-10) [30]	10	• PubMed hits: 147 • CD-RISC in total (25–10-2) available in more than 90 languages	• Paid questionnaire • Not recommended if content is reflected in other questionnaires	Optional
Connor Davidson Resilience Scale-2 (CD-RISC-2) [30]	2		• Low validity of German version • PubMed hits: 5	Rather not recommended
**Stress**				
Perceived Stress Questionnaire (PSQ-30) [31]	Classification of stressful life situations, 30	• Available in at least 5 languages	• Lengthy • PubMed hits: 5	Rather not recommended
Perceived Stress Scale (PSS-10) [32]	Experience of stress, 10	• PubMed hits: 525 • Available in at least 26 languages		Recommended
**Sense of coherence**				
Sense of coherence (SOC-13) [33]	13	• Linked to the concept of salutogenesis • PubMed hits: 233 • Available in at least 33 languages		Recommended
**Quality of life**				
Short Form Health Survey (SF-12) [34]	Quality of Life, 12	• PubMed hits: 2.150 • Available in at least 175 languages	• Paid questionnaire • Medical / physical focus	Rather not recommended
World Health Organization Quality of Life (WHOQOL-Bref) [35]	Quality of Life, 26	• Holistic, comprehensive • Suitable for healthy individuals • PubMed hits: 1.692 • Available in at least 78 languages	• Lengthy	Recommended
European Quality of Life (EQ-5D-5L) [36]	Quality of Life, in 5 dimensions, 25	• Simple, clear • Short • PubMed hits: 286 • Available in at least 150 languages	• Similar to SF-12 • Addresses patients • "Or" questions are methodologically questionable	Rather not recommended
**Wellbeing**				
Fragebogen zum allgemeinen habituellen Wohlbefinden (FAHW-12) [37]	General habitual well-being, 12		• Items are already covered in many other questionnaires (see quality of life, sense of coherence) • PubMed hits: 1 • Only available in German	Rather not recommended

The information on languages does not distinguish between languages, regional language types (e.g., British or American English) or dialects (e.g., British English still has a number of dialects)

#### Mindfulness

The expert panel decided to recommend the Mindful Attention and Awareness Scale (MAAS) [19] and the short form of the Self Compassion Scale (SCS) to assess mindfulness [20], as these complement each other well in terms of content. Furthermore, they were used more frequently in other evaluations than the Freiburg Questionnaire on Mindfulness (FMI) [17, 18]. The latter contains items on both self-love and mindfulness and therefore does not differentiate the constructs clearly enough.

#### Empathy

The Saarbrucken Personality Questionnaire (SPF) [22] was recommended for assessing empathy, as this is a German short form of the Interpersonal Reactivity Index (IRI) [21]. The SPF had good values for reliability, validity and item selectivity. Comprising 28 items, the IRI is lengthy, especially as strengthening empathy was not the primary objective of the course. Depending on the focus of the course, the Jefferson Scale of Physician Empathy (JSE) [24] was recommended as an option. It is limited to physician empathy. With 16 items, the Brief IRI (B-IRI) [23] is significantly shorter than the IRI or the SPF, but it was currently not available in a validated German version and was therefore not recommended.

#### Self-reflection

For the expert panel, self-reflection was a central component for changing one's own behavior and a prerequisite for dealing with stressful situations. It was therefore recommended to include the Groningen Reflection Ability Scale (GRAS) [25] in the evaluation. In terms of its content, it was well differentiated from other topics such as mindfulness and self-love. No other suitable questionnaires for self-reflection were found by the expert panel.

#### Self-efficacy

Self-efficacy was considered a very important topic by the expert panel. The General Self-Efficacy Scale (GSE) [26] was recommended, as it was frequently used in other evaluations. The shorter version, the GSE 6 [27], was not available free of charge. The Short Scale for Measuring General Self-efficacy Beliefs (ASKU) [28] had only been published recently and was recommended as an option, as it measures self-efficacy with only a few items.

#### Resilience

The Resilience Scale (RS-13) [29] was not recommended, as the content of the questionnaire strongly overlaps with the topic of self-efficacy. The terms “self-efficacy”, “sense of coherence” and “resilience” are difficult to differentiate from each other (see the discussion section for more detail). The Connor Davidson Resilience Scale (CD-RISC-10) [30] was used most frequently but was a paid questionnaire. It was recommended as optional if resilience should be assessed following the course objectives. The CD-RISC-2 [30] has low validity values and was therefore not recommended.

#### Stress

The Perceived Stress Scale (PSS-10) [32] was recommended because it measures the experience of stress with only 10 items and was frequently used in other evaluations. The Perceived Stress Questionnaire (PSQ-30) [31] is lengthy and therefore not recommended.

#### Sense of coherence

The expert panel recommended assessing the sense of coherence. It was seen as an important resource for dealing with challenging life situations [38]. The Sense of coherence (SOC 13) [33] is a suitable instrument that was frequently used.

#### Quality of life

The World Health Organization Quality of Life (WHOQOL-Bref) [35] assessment was recommended. It measures quality of life holistically and comprehensively and is suitable for assessing the quality of life of healthy participants. The items of the Short Form Health Survey (SF-12) [34], on the other hand, were more medically and physiologically oriented and therefore not recommended. The EuroQo (EQ-5D-5L) [36] uses "or" connecters within the items and was consequently not recommended due to methodological uncertainties.

#### Well-being

The assessment of well-being was not recommended, as many of the items in the questionnaire on general habitual well-being (FAHW-12) [37] were redundant, as they had already been included in other topics and questionnaires.

The questionnaires rated as "recommended" were then arranged in a meaningful order according to the learning objectives of the courses (Table 2). The questionnaire set was completed by all the experts of the panel to estimate the required time and the resulting burden on the respondents. The completion time for the questionnaire set was 20–30 min on average. In total, the questionnaire set comprised eight questionnaires with a total of 125 items and additional six questions on sociodemographic parameters such as age, sex, education, etc.

**Table 2:** Recommendation for the quantitative evaluation of MBM courses (basic evaluation)

Topic	Questionnaire	Items
Stress	Perceived Stress Scale (PSS-10) [32]	10
Quality of life	WHOQoL-Bref (WHOQoL-Bref) [35]	26
Mindfulness	Mindful Attention and Awareness Scale (MAAS) [19]	15
Self-efficacy	General Self-Efficacy Scale (GSE) [26]	10
Self-compassion	Self Compassion Scale (SCS) [20]	12
Empathy	Saarbrucken personality questionnaire (SPF) [22]	16
Self-reflection	Groningen Reflection Ability Scale (GRAS) [25]	23
Sense of coherence	Sense of coherence (SOC-13) [33]	13
Sociodemographics	Questions regarding age, sex, education, etc	6

In addition to the quantitative questionnaires, the expert panel also recommended a qualitative evaluation. In one of the underlying courses, data from focus groups was collected at the end of each course. The mixed methods evaluation yielded complementary and in-depth findings [3].

There was no consensus among the expert panel on further instruments of evaluation, such as the use of diaries or notes regarding the frequency of practice outside the courses. Writing a diary was welcomed by all experts as a stimulus for students to reflect, but a qualitative evaluation of the data would require considerable effort and time. The informative value of assessing the frequency of exercise outside the course seemed too low.

## Discussion

The basic evaluation compiled in Table 2 was the central result of the iterative consensus-based decision-making process by the expert panel. It reflects the previously defined learning objectives of the MBM course. The main emphasis was placed on the students' stress experience and quality of life, which are assessed using the PSS-10 and the WHOQOL-Bref. The MAAS, GSE, SCS and SOC will be used to evaluate whether the course has an impact on students' mindfulness and self-care. Empathy and the ability to self-reflect are prerequisites for successful communication. Hence, the evaluation results of the SPF and GRAS are possible indicators for a change in students' communication skills.

In addition to the basic evaluation with quantitative measures, a qualitative evaluation, e.g., in the form of group discussions, was recommended. It facilitates the identification of any other topics that were important to the course participants and that were not addressed in the questionnaires. Furthermore, qualitative results can help to better understand the quantitative results as well as supplement and deepen them [39].

Our recommendations must always be considered in the context of the individual course objectives. We would like to provide a guideline for the quantitative evaluation of students’ MBM courses with comparable objectives, e.g., stress reduction. In order to take the individuality of MBM courses at other universities into account, some questionnaires were recommended as "optional". Hence, each evaluation can be expanded individually, but the basic set of evaluation instruments remains the same.

### Strengths and limitations

One strength of the project is the interdisciplinary expertise of the expert panel. One of the medical experts works as a psychotherapist, while the other two have various additional qualifications in conventional, complementary and integrative medicine as well as in clinical epidemiology and qualitative research. The fourth expert is a medical scientist. Two of the experts work in clinical care. In addition, the expert panel represents two different MBM courses at two medical universities and thus contribute knowledge and experience from different courses. As scientists, the members of the panel have expertise in different research methods and many years of experience in the practice and teaching of MBM. Another strength is the iterative process.

The constructivist research paradigm of this work encouraged free thinking and exchange, creativity and situational object appropriateness. The collaboration in comparing and reflecting on the different course concepts also enabled an external perspective and thus a high level of abstraction. Another key strength is the practical applicability of the results.

The collaborative work between only two sites can be seen as a strength but also as a limitation. The relatively small panel of experts simplified working together, e.g., in finding appointments, developing a trusting and personal relationship and was the beginning of building a network. At the same time, the fact that other experts and their expertise were not included represents a limitation. Nonetheless, the expert panel decided against the involvement of more experts for organizational reasons and to avoid complexity. The questionnaires were selected through a structured evaluation as well as through discussions and consideration by consensus but not through a standardized procedure, e.g., by distributing points. This can be seen as a limitation as well as the fact that psychometric values of the questionnaires have not been considered, e.g., content validity. At the same time, the panel of experts felt that the analysis criteria were manageable so that weighing the advantages and disadvantages of the questionnaires was possible without scoring. Finding a consensus through discussion was seen as more important than a standardized allocation of points and comparing psychometric values of questionnaires, that are well known and widely used although not worldwide. A further limitation was the unsystematic search on further questionnaires, which was highly dependent on the experts’ thoroughness. Nevertheless, the inclusion of these additional questionnaires was beneficial.

In the present work, the authors addressed the evaluation of MBM courses for students and the corresponding instruments used, focusing on the evaluation of the course objectives. The assessment of the effectiveness of the courses, for which a control group would have been necessary, was not considered and should be object of future research.

### Resilience, self-efficacy and sense of coherence

As part of the iterative process, a number of conceptual discussions arose. These included the distinction between the topics of resilience, self-efficacy and sense of coherence. We present this discussion as an example and explain why we decided against the use and recommendation of a resilience questionnaire. Definitions of resilience are still inconsistent in the literature and the concept itself appears vague. The following question remains: Is resilience a process, an outcome or a personality trait [40]? Furthermore, the distinction between the terms “resilience” and “sense of coherence” as well as "resilience" and “self-efficacy” is not clear. This is also shown by the overlap in items of the respective questionnaires. The resilience scale measures what is represented in other questionnaires on psychological resilience, self-confidence and self-love, which are addressed in the basic evaluation by the SOC-13, GSE and SCS. Furthermore, there is a positive correlation between resilience and quality of life, life satisfaction, happiness/joy and well-being and a negative correlation with stress, anxiety and burnout. This should be taken into account when a questionnaire is selected to avoid unnecessary redundancies [41]. In summary, the discussion in the expert panel revealed that resilience is a complex construct that cannot be defined clearly and is therefore difficult to capture. Since the results of the resilience questionnaires would not have added any further informative value to the course evaluation, the expert panel decided against its inclusion.

### Evaluation of MBM courses

Smith et al. [42] published recommendations for the evaluation of MBM programs in the military healthcare setting. They described the development and implementation process of MBM courses in a very detailed manner and highlighted the importance of course evaluations. However, the authors did not give any specific recommendations on questionnaires but suggested the involvement of experts and scientists in the evaluation. There are many publications on evaluation results from MBM courses for students [3, 43–50]. The questionnaires used vary; however, stress was often assessed as the primary outcome. The sample sizes were usually small, which limits the interpretability of the results. Many times, the results were considered to be exploratory and their comparability was limited due to methodological differences.

## Conclusion

The iterative consensus-based decision-making process in the expert panel triggered discussions and reflections on the MBM courses that had been established for years. This led to refinement, deepening and improvement of the previous evaluation and learning objectives. The process resulted in a standardized, quantitative basic evaluation that can be used for MBM courses for students. An additional qualitative evaluation is recommended for a deeper and improved understanding of the results. We see the quantitative basic evaluation as an offer for other MBM courses, hoping that results can be pooled and compared across sites in the future. This would increase the informative value of the evaluation results. For future research, e.g. evaluations of MBM courses or randomized clinical studies in this field, we recommend to use the questionnaires we proposed and to critically evaluate them again in order to scrutinize their significance.

## Data Availability

The underlying data for this work comprise the questionnaires described in the article. These are available online free of charge or upon payment. Please see the respective references for more detail.

## Abbreviations

ASKU: Short Scale for Measuring General Self-efficacy Beliefs
B-IRI: Brief Interpersonal Reactivity Index
CD-RISC: Connor Davidson Resilience Scale
EQ: European Quality of Life
FAHW: Fragebogen zum allgemeinen habituellen Wohlbefinden
FMI: Freiburg Mindfulness Inventory
GRAS: Groningen Reflection Ability Scale
GSE: General Self-Efficacy Scale
IRI: Interpersonal Reactivity Index
JSE: Jefferson Scale of Physician Empathy
MAAS: Mindful Attention and Awareness Scale
MBM: Mind–body medicine
PSQ: Perceived Stress Questionnaire
PSS: Perceived Stress Scale
RS: Resilience Scale
SCS: Self Compassion Scale
SF: Short Form Health Survey
SOC: Sense of coherence
SPF: Saarbrucken personality questionnaire
WHOQOL: World Health Organization Quality of Life
